# Comparative Study of Reproductive Development in Wild and Captive-Reared Greater Amberjack *Seriola dumerili* (Risso, 1810)

**DOI:** 10.1371/journal.pone.0169645

**Published:** 2017-01-05

**Authors:** Rosa Zupa, Covadonga Rodríguez, Constantinos C. Mylonas, Hanna Rosenfeld, Ioannis Fakriadis, Maria Papadaki, José A. Pérez, Chrysovalentinos Pousis, Gualtiero Basilone, Aldo Corriero

**Affiliations:** 1 Department of Emergency and Organ Transplantation, Section of Veterinary Clinics and Animal Production, University of Bari Aldo Moro, Valenzano (Bari), Italy; 2 Departamento de Biología Animal, Edafología y Geología, Facultad de Ciencias, Universidad de La Laguna, La Laguna, Tenerife, Spain; 3 Institute of Marine Biology, Biotechnology and Aquaculture, Hellenic Center for Marine Research, Heraklion, Crete, Greece; 4 National Center for Mariculture, Israel Oceanographic and Limnological Research, Eilat, Israel; 5 Institute for Marine Coastal Environment, National Research Council, Capo Granitola (TP), Italy; Shanghai Ocean University, CHINA

## Abstract

The greater amberjack *Seriola dumerili* is a large teleost fish with rapid growth and excellent flesh quality, whose domestication represents an ambitious challenge for aquaculture. The occurrence of reproductive dysfunctions in greater amberjack reared in captivity was investigated by comparing reproductive development of wild and captive-reared individuals. Wild and captive-reared breeders were sampled in the Mediterranean Sea during three different phases of the reproductive cycle: early gametogenesis (EARLY, late April-early May), advanced gametogenesis (ADVANCED, late May-early June) and spawning (SPAWNING, late June-July). Fish reproductive state was evaluated using the gonado-somatic index (GSI), histological analysis of the gonads and determination of sex steroid levels in the plasma, and correlated with leptin expression in the liver and gonad biochemical composition. The GSI and sex steroid levels were lower in captive-reared than in wild fish. During the ADVANCED period, when the wild greater amberjack breeders were already in spawning condition, ovaries of captive-reared breeders showed extensive atresia of late vitellogenic oocytes and spermatogenic activity ceased in the testes of half of the examined males. During the SPAWNING period, all captive-reared fish had regressed gonads, while wild breeders still displayed reproductive activity. Liver leptin expression and gonad proximate composition of wild and captive greater amberjack were similar. However, the gonads of captive-reared fish showed different total polar lipid contents, as well as specific lipid classes and fatty acid profiles with respect to wild individuals. This study underlines the need for an improvement in rearing technology for this species, which should include minimum handling during the reproductive season and the formulation of a specific diet to overcome the observed gonadal decrements of phospholipids, DHA (22:6n-3) and ARA (20:4n-6), compared to wild breeders.

## Introduction

European consumer demand for more/new seafood products has been increasing over the last decade [1]. However, aquaculture plays only a minor role in the supply of high quality seafood to the European Union, providing only 10% of the total seafood consumption, whereas worldwide this value is >50% [2]. The limited variety of fresh and processed fish products coming from European aquaculture is considered an important bottleneck towards the expansion of this sector in Europe [3]. Domestication of new fish species [4] is considered as an effective tool to increase European aquaculture production and competitiveness, thus contributing to food security as well as to the reduction in fishing pressure on corresponding wild fish populations [5].

The greater amberjack *Seriola dumerili* (Risso, 1810) is a large coastal epibenthic and pelagic teleost fish with a wide geographical distribution, which includes the Indo-West Pacific Ocean [6], the Western Atlantic Ocean [7, 8], the Eastern Atlantic Ocean (from British to Moroccan coasts) and the Mediterranean Sea [9]. It is a gonochoric species with group-synchronous ovarian development and a multiple spawning pattern [10, 11], with a reproductive season between late spring to early summer in the Mediterranean region, and between later spring and early autumn in the Eastern Atlantic Ocean [12]. Greater amberjack is characterized by rapid growth, excellent flesh quality and has worldwide market appreciation [13, 14]. Its domestication represents an ambitious challenge for aquaculture, and has begun almost two decades ago [15–18]. However, large migratory, top predator fish do no adapt easily to captivity and when confined in sea cages or tanks they may exhibit important reproductive dysfunctions [11, 18–21]. Some of the reproductive dysfunctions of captive-reared greater amberjack have been overcome occasionally through the administration of exogenous reproductive hormones, such as human chorionic gonadotropin (hCG) [22–24] or gonadotropin releasing hormone agonists (GnRHa) [22, 23, 25, 26], while the occurrence of spontaneous spawning has been reported rarely [12]. However, the absence of a significant aquaculture industry for greater amberjack in Europe is testament to the lack of a reliable technology for broodstock management and reproduction control in captivity for this species [26].

In captivity, wild-caught greater amberjack often do not develop further than early vitellogenesis [18, 27] or if they do complete vitellogenesis, they fail to undergo oocyte maturation and require exogenous hormonal therapies to induce ovulation and spawning [26]. Different studies have been carried out in the Mediterranean Sea to investigate gametogenesis and describe the reproductive cycle either in wild [10, 28–30] or captive-reared greater amberjack [18, 24, 26]. However, so far no comparative study of the reproductive function of wild vs captive-reared greater amberjack has been conducted, in order to identify the extent and the points at which possible reproductive impairments occur in captivity. Moreover, although few studies exist on greater amberjack female gonad biochemical composition [31, 32], no information is available on male specimens, even though a close relationship between gonad composition–and more generally fish nutritional state- and reproductive success has been demonstrated widely [33–41]. More specifically, dietary fatty acids have proven to be very important in the reproduction of several fish species, including greater amberjack, since they determine gonad composition, affecting not only sperm and egg quality [31, 32, 37, 42–44], but also being involved in the synthesis of eicosanoids that are autocrine mediators in the reproductive process [38, 45–49]. Furthermore, the peptide hormone leptin plays a role in conveying signals of the energy stores to the central nervous system [50–55] and acts as a permissive factor for the onset of energy demanding processes such as reproduction [40, 56].

The objective of the present study was to identify the occurrence of the common reproductive dysfunctions during gametogenesis in greater amberjack caught as juveniles from the wild and reared to sexual maturity in captivity, through the comparative analysis of reproductive development during different times of the reproductive season. The reproductive state was assessed through gonad histological analysis, plasma sex steroid level determination, hepatic gene expression of leptin and gonad biochemical composition, including proximate composition, lipid classes and fatty acid profiles.

## Materials and Methods

### Ethical statement

For the present study, wild and captive-reared greater amberjack were sampled. Ethical approval was not required because this study did not fall within the obligations contained in the Italian decree n. 26 of 04 March 2014 regarding the permission to carry out research studies on experimental animals, as the fish came from a registered aquaculture facility and from commercial catches. The research did not involve any experiments on alive animals. Captive-reared fish originally came from the fishery at 0+ year of age, and were then reared at a registered aquaculture facility for 3 years, according to routine farming practices, before they were recruited for this study, sacrificed and sampled. Authors C.C.M. and Y.F. were involved in captive-reared fish killing and they declare that all relevant ethical safeguards were observed in relation to animal experimentation, and each fish was first anaesthetized with clove oil for 10 minutes and then painlessly sacrificed by decapitation. Wild greater amberjack were captured by the commercial purse seine fishing vessel “Graziella” authorized to catch pelagic fish by the port authority of Porto Empedocle (Agrigento, Italy). No specific permission was required because these fish were commercially caught during routine fishing operations, placed on ice by the fishermen and left to die. Immediately after death, those fish considered suitable for the present study were purchased and sampled on board. The greater amberjack is classified as “Least Concern” in the IUCN Red List of Threatened Species [57].

### Experimental animals, biometric data and sampling

A total of 33 (14 males and 19 females) wild and 24 (12 males and 12 females) captive-reared greater amberjack breeders were sampled at three different phases of the reproductive cycle that were determined according to the available literature [29, 30]: early gametogenesis (EARLY, late April-early May), advanced gametogenesis (ADVANCED, late May-early June) and spawning (SPAWNING, late June-July).

Wild fish were commercially caught around the Pelagie Islands (Sicily, Italy), during the fishing seasons of 2014 and 2015 and sampled on board immediately after death.

Captive-reared individuals were captured from the wild in 2011 in the area of Astakos (Ionian Sea, Greece). In September 2014, the fish were transferred to a sea cage of Argosaronikos Fishfarming S.A. (Salamina Island, Greece), where they were reared for two years according to standard farming practices. The fish were fed to apparent satiation every other day, during the first year with fresh fish, while during the year of the sampling the fish were switched to a commercial extruded broodstock diet (Vitalis-Cal, Skretting SA, Norway) (see S1 Table for diet proximate and fatty acid composition), as it is customary for aquaculture breeders of many species.

Before sampling, captive-reared fish were confined in a small cage area using a PVC curtain and then were tranquilized with about 0.01 ml l^-1^ clove oil (Roumpoulakis E.P.E., Greece) dissolved in ethanol at a 1:10 ratio. Then, they were gently directed into a PVC stretcher, brought on board of a service vessel, and anesthetized deeply with 0.03 ml l^-1^ clove oil. Subsequently, fish were sexed using a gonadal biopsy and a blood sample was obtained from the caudal vasculature using a heparinized syringe. Then the fish were euthanized by decapitation, were placed in crushed ice and transferred to the farm facility for further collection of biometric data and tissue samples.

For each fish, biometric data (fork length, FL, nearest cm; body mass, BM, nearest kg; gonad mass, GM, nearest g) were recorded (Tables 1 and 2). Blood, gonads and liver were collected and preserved according to specific protocols described below. The gonado-somatic index was calculated as GSI = 100 GM BM^-1^. During each sampling, the Sea Surface Temperature (SST, in °C) was recorded.

**Table 1:** Biometric data of wild and captive-reared greater amberjack females sampled during the reproductive season in the Mediterranean Sea, and Sea Surface Temperatures recorded at sampling sites.

Fish origin	Sampling Date	SST (°C)	FL (cm)	BM (kg)	GM (g)
**Early Gametogenesis (EARLY)**					
wild	01/05/2015	18.1	103	14	100
			103	15	200
			106	13	100
			112	19	200
			116	20	300
captive	24/04/2015	17.5	87	10	85
			96	14	125
			97	14	155
			100	14	160
**Advanced Gametogenesis (ADVANCED)**					
wild	31/05/2014	19.3	114	21	1600
			117	22	1650
captive	04/06/2015	20.0	97	13	335
			97	13	920
			101	12	660
			106	17	305
**Spawning (SPAWNING)**					
wild	29/06/2015	23.8	101	14	500
			109	16	700
			114	19	1000
	30/06/2014	23.4	95	12	450
			96	12	390
			97	12	450
			98	12	500
			99	11	500
			100	12	490
			100	12	400
			102	13	600
			104	14	950
captive	02/07/2015	25.5	92	8	95
			95	11	135
			96	12	130
			97	12	140

BM: Body Mass; FL: Fork Length; GM: Gonad Mass; SST: Sea Surface Temperature

**Table 2:** Biometric data of wild and captive-reared greater amberjack males sampled during the reproductive season in the Mediterranean Sea, and Sea Surface Temperatures recorded at sampling sites.

Fish origin	Sampling Date	SST (°C)	FL (cm)	BM (kg)	GM (g)
**Early gametogenesis (EARLY)**					
wild	01/05/2015	18.1	111	14	300
			112	20	450
			112	15	300
			113	19	400
			117	19	550
captive	24/04/2015	17.5	92	12	65
			94	12	60
			94	13	60
			101	15	95
**Advanced gametogenesis (ADVANCED)**					
wild	31/05/2014	19.3	99	14	1150
			102	13	650
			115	19	2200
			124	22	1900
captive	04/06/2015	20.0	90	9	370
			97	14	295
			98	13	600
			103	15	690
**Spawning (SPAWNING)**					
wild	29/06/2015	23.8	100	12	650
			102	14	700
			104	16	950
	30/06/2014	23.4	99	11	577
			100	11	400
captive	02/07/2015	25.5	91	10	70
			95	11	155
			96	13	140
			96	12	130

BM: body mass; FL: fork length; GM: gonad mass; SST: Sea Surface Temperature

### Histological analysis of greater amberjack ovaries and testes

For the histological analysis of greater amberjack ovaries and testes, 1-cm thick gonad slices were cut and fixed in Bouin's solution, dehydrated in ethanol, clarified in xylene and embedded in paraffin wax. Five-μm thick sections were then stained with haematoxylin-eosin, and Mallory’s trichrome. The assessment of the reproductive state of females was performed, according to Corriero et al. [19], on the basis of the most advanced oocyte stage, the occurrence of post-ovulatory (POFs) and atretic follicles. For the assessment of the male reproductive state, the type of spermatogenic cysts was recorded, and the amount of spermatozoa in the lumen of seminiferous lobules was subjectively evaluated [19].

### Sex-steroid plasma level measurement

Plasma was separated from the blood by centrifugation (5000 rpm for 5 minutes) and then was kept at -80°C until assayed for sex steroid determination. For the quantification of testosterone (T), 11-Ketotestosterone (11-KT) and 17,20β-dihydroxypren-4-en-3-one (17,20β-P) (a putative maturation-inducing steroid; MIS) in the plasma, already established and well-described enzyme-linked immunoassays (ELISA) were used [58–60] with some modifications, and using reagents from Cayman Chemical Company (USA). For the quantification of 17β-estradiol (E_2_), an ELISA kit was used (Cayman Chemical Company). For steroid extraction, 200 μl of plasma were extracted twice with 2 ml diethyl ether. Extraction was done by vigorous vortexing (Vibramax 110, Heidolph, Germany) for 3 min. After vortexing, samples were frozen for 10 min at -80°C and the supernatant organic phase was collected in new tubes and evaporated under a stream of nitrogen (Reacti-vap III, Pierce, Germany). Samples were reconstituted in reaction buffer for running in the ELISA.

### Cloning and sequencing of leptin cDNA and leptin real time-PCR

Small liver fragments were cut and kept in dry ice until they were transported to the laboratory, where they were stored at -80°C. In order to identify and clone the cDNA sequences encoding for leptin, total RNA from livers was extracted by the guanidiniumthiocyanate–phenol–chloroform extraction method using Bio-Tri RNA reagent (Bio Lab Ltd., Jerusalem, Israel). One microgram of DNAse treated total RNA was reverse transcribed with random primers using the High Capacity cDNA Reverse Transcriptase kit (Applied Biosystems, Branchburg, NJ, USA) according to manufacturer’s protocol. For initial cloning, PCR amplification was conducted using degenerate primers that were designed according to the most conserved regions across Perciforms (Table 3).

**Table 3:** (a) Primer list used to clone greater amberjack leptin; (b) Comparable sequence from closely related species for primer design; (c) Primers for greater amberjack real-time PCR.

**(a)**	**Target gene**	**Sequence**
	Seriola Lep F1	GAAATCAAAAGTGAAATGGATGG
	Seriola Lep F3	CCAGGTCCCTCCTGGCCTGAC
	Seriola Lep R3	TTGACCTGRGWGACYCCRTY
**(b)**	**Target gene**	**Species—Accession number**
	Leptin	*Atlantic bluefin tuna*—HQ288053
**(c)**	**Target gene**	**Real time-PCR Primers**
	leptin FOR	CCGTTAAGGGTGTCAGAGA
	leptin REV	TTCCAGGTCCCTGTTGGTC
	β-actin FOR	CCCTGTCCTGCTCACAGAGG
	β-actin REV	CAAGTCCAGACGCAGGATGG

The obtained PCR products were purified with QIAquick PCR Purifcation Kit (QIAGENE, Hilden, Germany) cloned into pGEM^®^-T Easy vector (Promega, Madison, WI), and sequenced with ABIPRISM^®^ 3100 Genetic Analyzer (Applied Biosystems, Foster City, CA) at the DNA Biological Services, Tel Aviv University, Israel. The nucleotide sequences were translated using the Just Bio translator program (www.justbio.com) and their identities confirmed using the BLAST algorithm (Blastp) of the National Center for Biotechnology Information (Bethesda, MD).

Once the greater amberjack homologous sequences were obtained, leptin (Lep) and β-actin specific primers were designed (Table 3) employing the Primer3 software [61] and used to establish quantitative real-time PCR (qPCR) for gene expression analysis. Total RNA was obtained from liver using the RNeasy Mini Kit (Qiagen) as described by the manufacturer. Reverse transcription of 1000 ng of total RNA was performed using SuperScript III Reverse Transcriptase (Invitrogen^®^) and diluted cDNA (1:10) was used in all following qPCR reactions. The qRT-PCR experiments were carried out in triplicate using the QuantStudio^™^ 7 Flex System (Applied Biosystems^®^, Thermo Fisher SCIENTIFIC, Milan, Italy) using 1μl of diluted cDNA as template for each reaction with SYBR Green PCR Master Mix (Bio-Rad). The presence of a single amplicon was verified using a melting curve run following the PCR. No template controls were included as negative controls for each primer pair. The quantification of the β-actin gene was used as the endogenous control. Amplification parameters were as follows: hot start at 95°C for 15 min; 40 amplification cycles (95°C for 15 sec, 60°C for 30 sec, 72°C for 30 sec); dissociation curve step (95°C for 15 sec, 60°C for 15 sec, 95°C for 15 sec). Fluorescence raw data were exported from the QuantStudio Real Time PCR software (Applied Biosystems^®^, Thermo Fisher SCIENTIFIC) and analysed with the DART-PCR Excel workbook [62]. Actual amplification efficiency values (E) for each amplicon were used to correct Cq values before analysing these data by the ΔCq method to compare relative expression results. Gene expression levels were calculated by: relative expression = 2^-ΔΔCt^ [63].

### Gonad proximate composition, lipid classes and fatty acid profiles

To evaluate gonad biochemical composition, pieces of ovaries and testes were cut and kept in dry ice until they were transported to the laboratory, where they were immediately stored at -80°C until analysis. Dry matter and protein contents were calculated using the methods of analysis of the Association of Official Analytical Chemists [64]. Moisture content was determined in 500-mg samples by thermal drying in an oven at 110°C, until constant weight. Protein was determined by sample digestion according to the Kjeldahl method. Total lipid (TL) was extracted by sample homogenization in chloroform/methanol (2:1, v/v) according to the method of Folch et al. [65]. The organic solvent was evaporated under a stream of nitrogen and the lipid content was determined gravimetrically [66] and stored in chloroform/methanol (2:1), containing 0.01% butylated hydroxytoluene (BHT). Analysis of lipid class (LC) composition was performed by one-dimensional double development high performance thin layer chromatography (HPTLC; Merk, Darmstadt, Germany), and methyl acetate/isopropanol/chloroform/methanol/0.25% (w/v) KCl (5: 5: 5: 2: 1.8, by volume) used as developing solvent system for the polar lipid classes and isohexane/diethyl ether/acetic acid (22.5: 2.5: 0.25, by volume), for the neutral lipid separation. Lipid classes were visualized by charring at 160°C for 15 min after spraying with 3% (w/v) aqueous cupric acetate containing 8% (v/v) phosphoric acid, and quantified by scanning densitometry using a dual-wavelength flying spot scanner Shimadzu CS-9001PC (Shimadzu, Duisburg, Germany) [67]. To determine the fatty acid profiles, TL extracts were subjected to acid-catalysed transmethylation with 1% sulphuric acid (v/v) in methanol. The resultant fatty acid methyl esters (FAME) and dimethyl acetals (DMA) were extracted using isohexane: diethylether (1:1 by volume) and purified by TLC using isohexane/diethyl ether/acetic acid (90:10:1, by volume) as developing system [66]. Fatty acid methyl esters were separated and quantified using a TRACE-GC Ultra gas chromatograph (Thermo Electron Corp., Waltham, MA, USA) equipped with an on-column injector, a flame ionization detector and a fused silica capillary column, Supelcowax TM 10 (30 m x 0.32 mm I.D. x 0.25 μm; Sigma-Aldrich, Madrid, Spain). Helium was used as carrier gas and temperature programming was 50–150°C at 40°C min^-1^ slope, then from 150 to 200°C at 2°C min^-1^, to 214°C at 1°C min^-1^ and, finally, to 230°C at 40°C min^-1^. Individual FAME and DMA were identified by reference to authentic standards, and further confirmation of FAMEs and DMAs identity was carried out by GC-MS (DSQ II; Thermo Electron Corp.).

### Statistical analysis

Differences in GSI, sex steroid concentrations, leptin qRT-PCR and biochemical composition mean values between the following pair of groups were assessed by a two tailed Student’s t-test: wild specimens sampled in consecutive phases of the reproductive cycle; captive-reared specimens sampled in consecutive phases of the reproductive cycle; wild *vs* captive-reared specimens sampled in the same phase of the reproductive cycle. Normality and homogeneity of variance were confirmed and percentage data were arcsine transformed prior to analysis.

All the results are presented as means ± SE; the statistical probability significance was established at the *P* ≤ 0.05 level. The statistical analyses were performed using the SPSS 17.0 software package (IBM Corp., New York, USA) for Windows.

## Results

### Female GSI, gonad histological analysis and sex-steroid plasma levels

**Fig 1:**
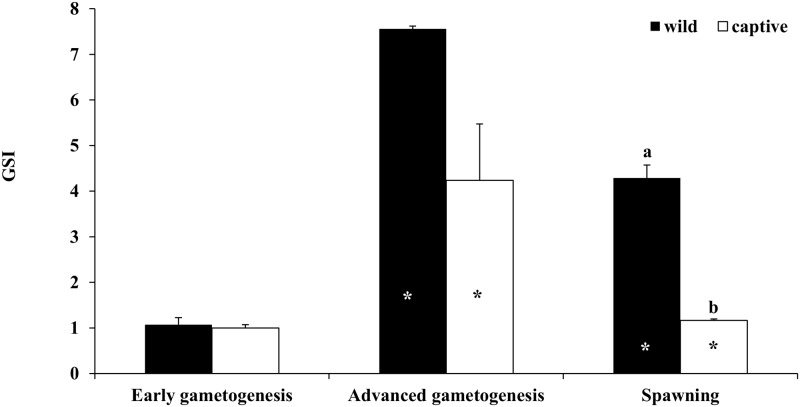
Mean (± SE) gonado-somatic index (GSI) of wild and captive-reared greater amberjack females sampled in three phases of the reproductive season. White and black asterisks indicate statistically significant differences versus the preceding phase in wild specimens and in captive-reared specimens, respectively. Different letters indicate significant differences between wild and captive-reared individuals in the same phase of the reproductive cycle (*P* < 0.05).

In both wild and captive-reared individuals, GSI values showed a significant increase from the EARLY to the ADVANCED phase, and a significant decrease during SPAWNING (Fig 1). The GSI values were similar between captive and wild females during the EARLY and ADVANCED phases, while they were significantly higher in wild fish during SPAWNING.

**Fig 2:**
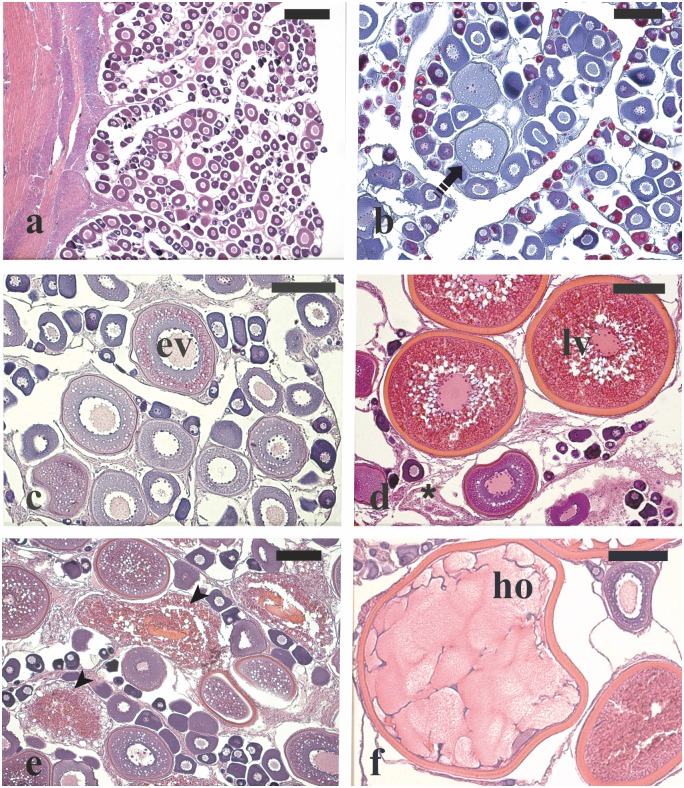
Micrographs of ovary sections from female greater amberjack sampled in three different phases of the reproductive season. (a) Wild individual sampled on 1 May showing perinucleolar oocytes as the most advanced stage in the ovary. (b) Cortical alveoli oocytes in the ovary of a wild specimen captured on 1 May 2015. (c) Early vitellogenic oocytes in the ovary of a wild individual sampled on 1 May 2015. (d) Late vitellogenic oocytes together with post-ovulatory follicles from a wild spawning fish caught on 31 May 2014. (e) Extensive atresia of late vitellogenenic follicles in a captive-reared specimen sampled on 4 June 2015. (f) Hydrated oocyte from a spawning wild fish sampled on 30 June 2014. Haematoxylin-eosin staining in (a), (c), (d), (e) and Mallory’s trichrome staining in (b). Magnification bars = 300 μm in (a) and 150 μm in (b)-(f). Arrowhead: atretic late vitellogenic follicle; asterisk: post-ovulatory follicle; dashed arrow: cortical alveoli stage oocyte; ev: oocyte in early vitellogenesis stage; ho: hydrated oocyte; lv: oocyte in late vitellogenesis stage.

During the EARLY phase in wild females, one individual had perinucleolar oocytes as the most advanced oocyte stage (Fig 2a), two had oocytes at the cortical alveoli stage (Fig 2b), and two exhibited early vitellogenic oocytes (Fig 2c). Among the four captive-reared females, one had ovaries with perinucleolar oocytes and three showed few early vitellogenic oocytes. In the ADVANCED phase, the two wild females showed oocytes at the late vitellogenic stage and POFs, a sign of recent spawning (Fig 2d). All four captive-reared females had oocytes at late vitellogenesis and three of them displayed major α atresia (> 50% of vitellogenic oocytes in α atresia) (Fig 2e). In the SPAWNING phase, among the 12 wild fish sampled, 10 had late vitellogenic oocytes together with POFs, and two individuals showed hydrated oocytes (Fig 2f). Among the four captive-reared females, three showed ovaries with late vitellogenic oocytes undergoing extensive atresia and one showed only perinucleolar oocytes, indicating that all these animals were in a regressed condition.

**Fig 3:**
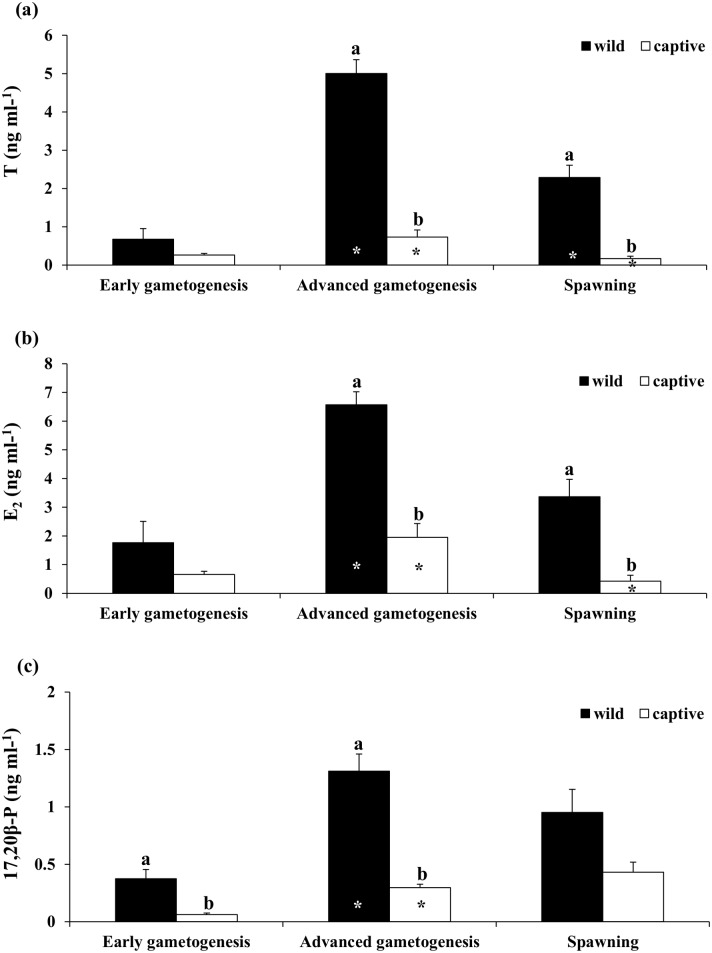
Mean (± SE) plasma (a) Testosterone (T), (b) 17-β Estradiol (E_2_) and (c) 17,20β-P plasma in wild and captive-reared greater amberjack females at three phases of the reproductive season. White and black asterisks indicate statistically significant differences versus the preceding phase in wild specimens and in captive-reared specimens, respectively. Different letters indicate significant differences between wild and captive-reared individuals in the same phase of the reproductive cycle (*P* < 0.05).

In wild females, plasma levels of T, E_2_ and 17,20β-P increased significantly from the EARLY to the ADVANCED phase, while in the case of T they decreased during SPAWNING (Fig 3). In captive-reared females, both T and E_2_ increased significantly from the EARLY to the ADVANCED phase and then decreased during SPAWNING, while plasma 17,20β-P did not decrease significantly at the SPAWNING stage. Significantly higher T and E_2_ plasma levels were found in wild compared to captive-reared animals at the ADVANCED and SPAWNING phases. Plasma 17,20β-P levels were significantly higher in wild compared to captive-reared fish during the EARLY and ADVANCED phases.

### Male GSI, gonad histological analysis and sex-steroid plasma levels

**Fig 4:**
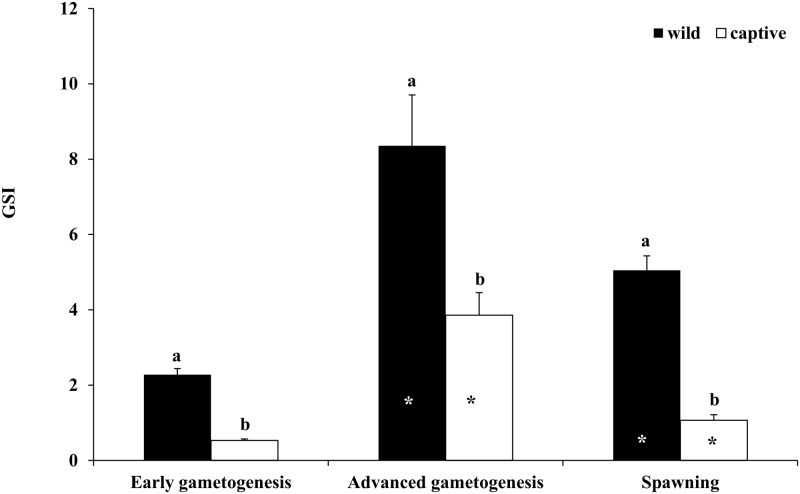
Mean (± SE) gonado-somatic index (GSI) of wild and captive greater amberjack males sampled in three phases of the reproductive season. White and black asterisks indicate statistically significant differences versus the preceding phase in wild specimens and in captive-reared specimens, respectively. Different letters indicate significant differences between wild and captive-reared individuals in the same phase of the reproductive cycle (*P* < 0.05).

Both in wild and captive-reared males, GSI showed a significant increase from EARLY to ADVANCED, followed by a decrease during SPAWNING (Fig 4). In all the three considered phases, GSI was significantly higher in wild than in captive-reared males.

**Fig 5:**
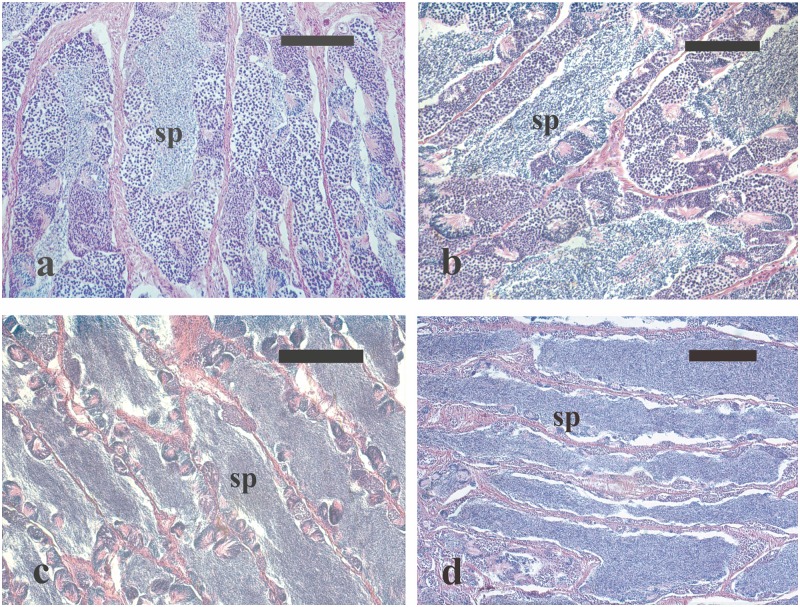
Micrographs of testes sections from male greater amberjack sampled in three different phases of the reproductive season. (a) Testis section from a wild individual sampled on 1 May showing the presence of all stages of spermatogenesis in the germinal epithelium and a limited amount of luminal spermatozoa. (b) Testis section from a wild fish caught on 31 May 2014, showing all stages of spermatogenesis as well as large amount of luminal spermatozoa. (c) Testis section from a captive-reared fish sampled on 4 June 2015 showing an arrested spermatogenesis state, with residual sperm cysts in the germinal epithelium and abundant spermatozoa in the lumen of seminiferous lobules. (d) Testis sections from a captive-reared specimen caught on 2 July 2015 showing a moderate amount of spermatozoa in the lumen of seminiferous lobules. Haematoxylin-eosin staining. Magnification bars = 100 μm in (a) and (b), 200 μm in (c) and (d). sp: spermatozoa in the lumina of seminiferous lobules.

The testes of the five wild males caught during the EARLY period contained germ cells in all spermatogenic stages, as well as spermatozoa in the seminiferous lobules (Fig 5a). The histological appearance of the testes of the four captive-reared males sampled in the same phase was similar to that of wild males. However, the former displayed a lower amount of luminal spermatozoa. In the ADVANCED phase, all the four wild males had all stages of spermatogenesis in the germinal epithelium as well as large amount of luminal spermatozoa (Fig 5b). Among the four captive-reared males sampled in this phase, two were in active spermatogenesis, whereas the other two had ceased their spermatogenic activity, having only residual sperm cysts in the germinal epithelium and abundant spermatozoa in the lumen of seminiferous lobules (Fig 5c).

In the SPAWNING phase, four wild males showed all stages of spermatogenesis together with large amount of spermatozoa in the lumen of seminiferous lobules and one was partially spent, showing rare spermatocysts and residual spermatozoa in the lumen of seminiferous lobules. All the four captive-reared males sampled during this phase had ceased their spermatogenic activity, still showing a moderate amount of spermatozoa in the lumen of seminiferous lobules (Fig 5d).

**Fig 6:**
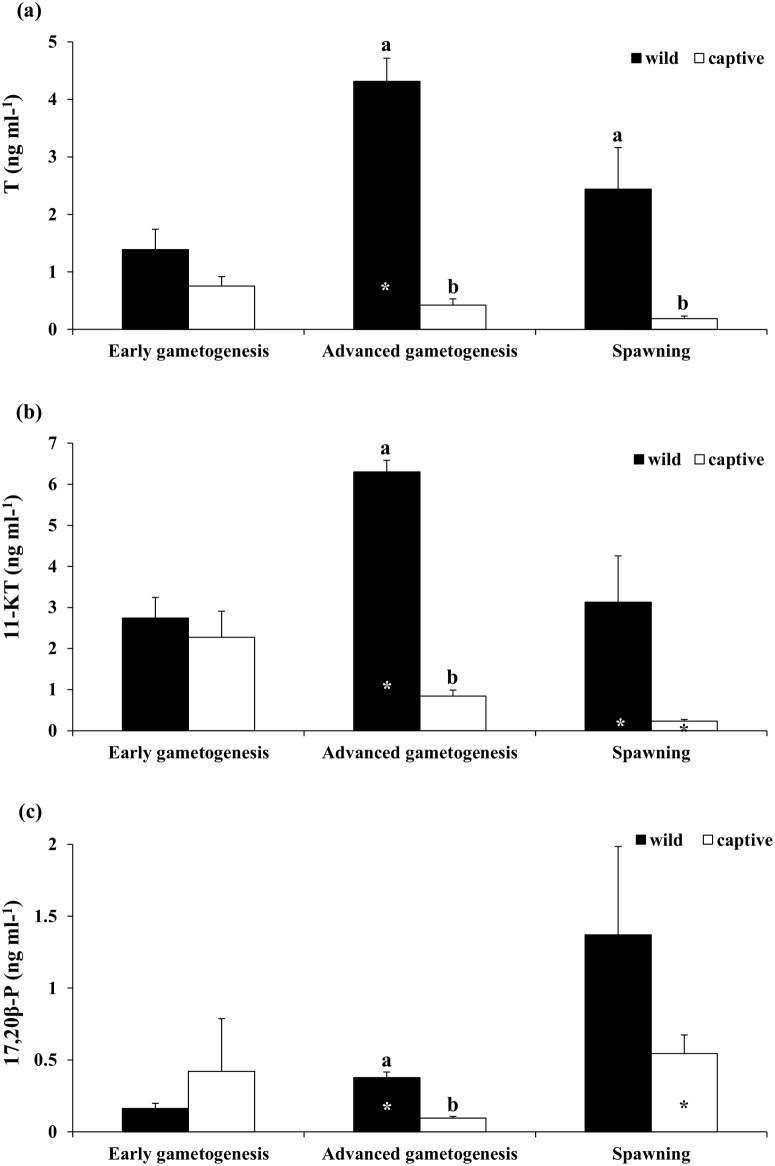
Mean (± SE) plasma (a) Testosterone (T), (b) 11-Ketotestosterone (11-KT) and (c) 17,20β-P in wild and captive-reared greater amberjack males at three phases of the reproductive season. White and black asterisks indicate statistically significant differences versus the preceding phase in wild specimens and in captive-reared specimens, respectively. Different letters indicate significant differences between wild and captive-reared individuals in the same phase of the reproductive cycle (*P* < 0.05).

In wild males, T and 11-KT plasma levels increased significantly from the EARLY to the ADVANCED phase and decreased thereafter in case of only 11-KT (Fig 6). Plasma levels of 17,20β-P showed a significant increase from the EARLY to the ADVANCED phase, and the same trend was observed at the SPAWNING phase, even though a significant difference was not found (Fig 6). In captive-reared fish, both T and 11-KT plasma levels showed a decreasing trend from the EARLY to the SPAWNING phase, whereas plasma 17,20β-P levels showed a significant increase from the ADVANCED to the SPAWNING phase (Fig 6). In general, plasma levels of all three analysed steroids were higher in wild than in captive-reared fish.

### Liver leptin gene expression

The partial cDNA sequence of the greater amberjack leptin (131 base pairs long; S1 Fig) was found to encompass the typifying alpha-helix domains (i.e., Helix B and 5' end of Helix C), and to share a large degree of homology (90%) with cognate sequence derived from the Chinese perch *Siniperca chuatsi* (S2 Table).

**Fig 7:**
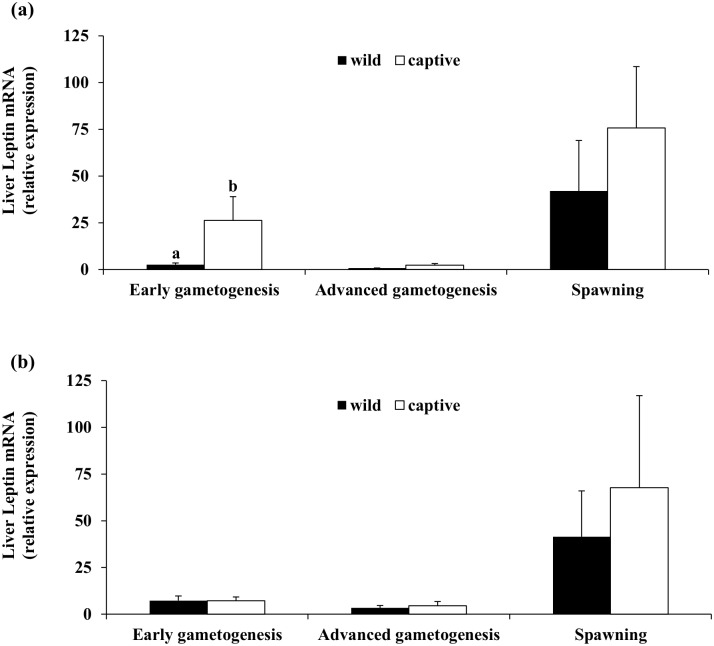
Mean (± SE) transcription levels of liver leptin in three phases of the reproductive season of wild and captive-reared greater amberjack. (a) Females and (b) Males. Different letters above bars indicate statistically different transcription levels between wild and captive-reared specimens within the same sampling phase (*P* < 0.05).

Quantitative real-time PCR (qRT-PCR) analysis of liver leptin mRNA (Fig 7) demonstrated that transcript levels in both wild and captive-reared fish were minimal during the ADVANCED phase and maximal at SPAWNING. Significant differences between wild and captive-reared specimens were observed only in females during the EARLY phase (Fig 7a), when higher liver leptin mRNA levels were observed in captive individuals.

### Assessment of gonad biochemical composition

#### Gonad proximate composition

The analysis of ovary proximate composition during the reproductive cycle of wild and captive-reared fish showed a clear moisture reduction, associated to a significant protein and lipid increase at the ADVANCED compared to the EARLY phase, followed by a trend to recuperate the original values at SPAWNING (Table 4). In the testes, moisture contents slightly decreased from the ADVANCED to the SPAWNING phase in captive fish, whereas protein levels rose between the EARLY and ADVANCED phases (Table 4).

**Table 4:** Mean (± SE) levels of moisture, total lipids and total proteins of gonads from wild and captive-reared greater amberjack sampled at three different phases of the reproductive cycle.

	Early Gametogenesis (EARLY)		Advanced Gametogenesis (ADVANCED)		Spawning (SPAWNING)	
	Wild	Captive	Wild	Captive	Wild	Captive
***Ovaries***						
**Moisture (%)**	81.4±0.3	80.7±0.3	68.1±0.3 *	71.5±1.7 *	74.0±0.8 *	78.2±1.4 *, †
**TL (%ww)**	1.5±0.1	1.5±0.2	6.6±0.8 *	5.5±0.5 *	4.5±0.2 *	3.6±0.6 *
**Protein (%ww)**	15.7±0.4	15.8±0.2	20.6±0.3 *	19.3±1.2 *	18.0±0.8	15.4±0.7 *,†
***Testes***						
**Moisture (%)**	83.9±0.1	83.7±0.1	84.7±0.1	83.3±0.2 †	84.9±0.3	81.8±0.6 *, †
**TL (%ww)**	2.1±0.2	2.3±0.2	2.0±0.4	2.5±0.2	1.8±0.1	2.1±0.2
**Protein (%ww)**	12.6±0.2	12.5±0.1	12.4±0.2	14.2±0.4 *, †	12.3±0.3	15.1±0.7 †

Asterisks indicate statistically significant differences versus the preceding phase in wild specimens and in captive-reared specimens distinctly,

^†^ denote significant differences between wild and captive-reared individuals at the same phase of the reproductive cycle. TL: total lipids; ww: wet weight.

Captivity was not associated with major changes in the general proximate composition of the gonads, with the exception of higher moisture and lower protein contents in the ovaries of captive-reared fish during SPAWNING, as well as lower humidity and higher protein levels in testes of captive-reared fish during the ADVANCED and SPAWNING phases (Table 4).

#### Gonad lipid classes and fatty acid composition

Significant differences in the main lipid classes (Table 5) and fatty acid compositions (Table 6) were found in wild and captive-reared gonads of greater amberjack throughout the reproductive cycle.

**Table 5:** Mean values (± SE) of main lipid classes (% of total lipids) of gonads from wild and captive-reared greater amberjack sampled at three different phases of the reproductive cycle.

	Early Gametogenesis (EARLY)		Advanced Gametogenesis (ADVANCED)		Spawning (SPAWNING)	
	Wild	Captive	Wild	Captive	Wild	Captive
***Ovaries***						
**PC**	21.6±0.7	18.4±1.1 †	18.5±1.0	17.7±0.9	18.3±0.7	17.3±0.3
**PS**	4.5±0.5	4.5±0.8	0.8±0.1 *	1.5±0.2 *	1.4±0.1	3.0±0.4 *, †
**PI**	4.9±0.3	3.8±0.3	1.8±0.1 *	2.9±0.2	2.5±0.1	3.4±0.3 †
**PE**	12.2±0.4	11.8±1.1	5.2±0.6 *	5.9±0.3 *	6.2±0.2	7.9±0.7 *, †
**TAG**	21.0±0.7	26.3±4.3	28.2±0.2 *	26.4±0.9	21.5±0.6	21.3±1.3
**TPL**	47.8±1.2	43.8±3.5	28.9±2.0 *	30.8±0.3 *	31.1±1.1	35.6±1.9 *, †
***Testes***						
**PC**	27.6±1.3	20.5±0.9 †	25.8±0.5	22.9±1.5	27.8±0.4	23.1±0.4 †
**PS**	7.8±0.5	4.6±0.4 †	12.3±0.4 *	8.8±0.6 *,†	9.7±0.8	8.8±0.4
**PI**	5.9±0.2	5.0±0.5	1.7±0.3 *	6.8±0.4 †	5.6±1.4 *	7.1±0.1
**PE**	20.0±0.4	14.1±1.0 †	22.1±0.3	21.2±1.3 *	21.4±0.3	21.9±0.7
**TAG**	8.7±0.9	28.9±4.3 †	3.0±0.5 *	2.8±0.8 *	1.8±0.3	4.4±0.8 †
**TPL**	64.6±1.9	47.4±2.6 †	65.2±0.2	63.8±3.7 *	67.6±1.5	65.7±0.8

Asterisks indicate statistically significant differences versus the preceding phase in wild specimens and in captive-reared specimens distinctly,

^†^ denote significant differences between wild and captive-reared individuals at the same phase of the reproductive cycle. PC, phosphatidylcholine; PS, phosphatidylserine; PI, phosphatidylinositol; PE, phosphatidylethanolamine; TAG, triacylglycerols; TPL, total polar lipids.

**Table 6:** Mean values (± SE) of main fatty acids of gonads from wild and captive-reared greater amberjack sampled at three different phases of the reproductive cycle.

	Early gametogénesis (EARLY)		Advanced gametogénesis (ADVANCED)		Spawning (SPAWNING)	
	Wild	Captive	Wild	Captive	Wild	Captive
***Ovaries***						
**LA**	1.0±0.1	6.5±0.3 †	2.2±0.3 *	10.1±0.3 *, †	1.8±0.1	8.1±1.1 *, †
**ARA**	5.9±0.4	3.4±0.6 †	3.4±0.2 *	2.1±0.2 *, †	5.1±0.2 *	4.0±0.3 *
**EPA**	3.9±0.2	5.3±0.2 †	3.4±0.1	5.1±0.7 †	4.2±0.1	4.3±0.3
**DHA**	27.3±0.8	19.4±1.9 †	23.8±2.0	21.1±0.8	23.0±0.5	23.3±1.4
**ARA/EPA**	1.6±0.2	0.6±0.1 †	1.0±0.1	0.4±0.1 †	1.2±0.1	1.0±0.1 *
**DHA/EPA**	7.1±0.5	3.6±0.2 †	6.9±0.3	4.4±0.7 †	5.5±0.2	5.8±0.3
***Testes***						
**LA**	1.1±0.1	7.1±0.4 †	0.8±0.1	5.0±0.6 *, †	1.0±0.1	5.8±1.0 †
**ARA**	4.1±0.4	2.3±0.2 †	4.3±0.2	2.7±0.2 †	5.4±0.4	5.0±0.7 *
**EPA**	3.7±0.2	4.6±0.4	2.9±0.2	4.8±0.5 †	2.8±0.2	3.8±0.4 †
**DHA**	26.2±1.6	18.1±1.5 †	32.9±1.0 *	27.0±1.8 *, †	26.9±0.9 *	24.6±1.8
**ARA/EPA**	1.1±0.1	0.5±0.0 †	1.5±0.2	0.6±0.0 †	2.0±0.3	1.5±0.4 *
**DHA/EPA**	7.1±0.3	3.9±0.1 †	11.3±0.5 *	5.7±0.3 *, †	10.0±0.9	6.7±0.3 †

Asterisks indicate statistically significant differences versus the preceding phase in wild specimens and in captive-reared specimens distinctly,

^†^ denote significant differences between wild and captive-reared individuals at the same phase of the reproductive cycle. Individual fatty acids are expressed as percentage of total fatty acids. LA, linoleic acid, 18:2n-6; ARA, arachidonic acid, 20:4n-6; EPA, eicosapentaenoic acid, 20:5n-3; DHA, docosahexaenoic acid, 22:6n-3.

In wild fish ovaries, the lipid class composition dramatically varied between the EARLY and ADVANCED phases, where a decline in individual polar lipid classes and a rise in triacylglycerol (TAG) proportions was registered. Variations in the ovaries of the captive fish were more moderate with significant reductions between the EARLY and ADVANCED phases in phosphatidylserine (PS) and phosphatidylethanolamine (PE) levels, which returned to higher values thereafter. As it is shown in Table 5, evolution of the lipid class composition in wild fish testes differed markedly from that of ovaries, highlighting a significant increment of PS and a reduction of phosphatidylinositol (PI) and TAG between the EARLY and ADVANCED phases. From the ADVANCED to the SPAWNING phase, only PI varied significantly towards recuperation. Gonad levels of TAG were three times higher at the EARLY phase in testis of captive-reared fish than in wild specimens, leading to a lower relative proportion of phosphatidylcholine (PC), PS and PE. In addition, TAG exhibited a 10-fold reduction in the testes of captive fish from the EARLY to the ADVANCED phase, whereas PI levels did not decrease as in wild specimens, but tended to rise.

Significant differences in gonad fatty acid composition were also found between wild and captive specimens, particularly during the EARLY and ADVANCED phases, with both ovaries and testes of captive fish displaying around 30 and 40% less docosahexaenoic acid (DHA) and arachidonic acid (ARA), respectively (Table 6), and, as a consequence, a significantly lower DHA/eicosapentaenoic acid (EPA) and ARA/EPA ratios. Moreover, captive-reared fish gonads also presented higher contents of octadecadienoic acid (linoleic acid, 18:2n-6) (LA). Both in wild and captive-reared specimens, testis DHA content increased significantly between the EARLY and ADVANCED phases, followed by a decrease to initial values at the SPAWNING period only in wild fish (Table 6).

## Discussion

In the present work, the reproductive state of greater amberjack was compared between wild and captive-reared breeders during different phases of their reproductive cycle in the Mediterranean Sea, in an attempt to assess the effects of rearing in captivity on reproductive maturation. Fishery data suggest that greater amberjack aggregate in shallow water for reproduction from May to July, when they become vulnerable to the purse-seine fishery [68, 69]. Spawning occurs mainly between June and early July, when females with ovaries containing hydrated oocytes and post-ovulatory follicles have been found [29]. Therefore, the sampling in the present study was carried out in three different periods of the reproductive cycle that were considered *a priori* to coincide with early gametogenesis (late April-early May), advanced gametogenesis (late May-early June) and spawning (late June-early July).

All of the wild and captive-reared fish used in the present study were beyond their first sexual maturity, as determined for the Mediterranean greater amberjack by Kožul et al. [70]. The available data on greater amberjack first sexual maturity in the Mediterranean Sea are scarce and somehow contradictory, and refer to wild fish sampled in the Pelagie Islands [10], Gulf of Gabes (Tunisia) [30] and Adriatic Sea [70], and to captive fish reared in outdoor tanks in Sicily [18]. In the Adriatic Sea, 80% of age class 4 (93–106 cm total length, TL) females were reproductively active and 100% maturity was reached at 5 years of age (107–119 cm TL); similarly, all males with TL > 107 cm sampled in the Adriatic Sea were reported to be sexually mature [70]. In the present study, FL was used as fish body length measure and the recorded sizes ranged from 95 to 124 and from 87 to 106 cm for wild and captive-reared specimens, respectively. According to the TL-FL correlations provided for male and female greater amberjack from the eastern Mediterranean Sea by Sley et al. [30], all the fish of the present study were > 107 cm TL except the smallest captive-reared fish that was 106 cm TL.

Gonad development of wild greater amberjack during the sampling period was well described by the GSI, increasing from early May to late May and decreasing thereafter. The histological analysis of wild greater amberjack gonads showed that at the beginning of May ovaries exhibited early vitellogenic oocytes, and testes contained germ cells at all stages of spermatogenesis, including luminal spermatozoa. In late May, females exhibited fully vitellogenic oocytes together with post-ovulatory follicles in their ovaries, and males had testes in full spermatogenesis, with all the spermatogenic stages in the germinal epithelium and large amounts of luminal spermatozoa. In late June, fish were still in reproductive condition, with late vitellogenic and hydrated oocytes present in the ovaries and with most males still exhibiting active spermatogenesis and plenty of luminal spermatozoa. The evolution of GSI of wild and captive-reared greater amberjack during the reproductive season was similar; however, in general GSI values were significantly lower in captive-reared fish. Ovaries and testes of captive-reared fish sampled during late April (EARLY) showed an overall maturity stage comparable to that of wild individuals sampled in the same period (early May). However, the subsequent gonad maturation phase appeared to be seriously impaired in captive fish, since during the second sampling campaign (ADVANCED) an extensive atresia of late vitellogenic oocytes affected the ovaries, and half of the sampled males had already ceased their spermatogenic activity. In late June (SPAWNING), the wild greater amberjack population was still in spawning condition, whereas in the same period all fish reared in captivity showed regressed gonads.

The trend of GSI and the histological data on gonad maturation of wild and captive reared greater amberjack were in close agreement with their sex steroid plasma concentrations. All the androgens examined in the present study were constantly lower in captive than in wild fish. In males, similar concentrations of 11-KT, but higher of T during the EARLY and ADVANCED phases, were observed in a study with wild greater amberjack sampled in Pelagie Islands, showing a peak in late May-early June [29]. As for many male teleost fish [71–73], 11-KT proved to be the prominent androgen in greater amberjack, always having higher plasma levels than T. Regarding 17,20β-P, in wild male fish it followed the increase of GSI from the EARLY to ADVANCED phase, in agreement with its well-known function in enhancing sperm production and volume [74, 75]. On the other hand, in captive-reared males in the present study, 17,20β-P plasma levels showed a surprising increase between the ADVANCED and SPAWNING phases, in concomitance with a GSI decrease and testis regression. The same was found to happen in tench *Tinca tinca*, where an inexplicable peak of 17,20β-P found in males with regressed gonads [76]. The existing literature on 17,20β-P in greater amberjack and other related species has been limited mostly to females [29, 77, 78]; the only study including males, carried out on the congener yellowtail kingfish *Seriola lalandi*, failed to find any difference in the 17,20β-P values between the different reproductive stages [79]. The generally low 17,20β-P values observed both in the present study and in other studies on greater amberjack and related species [29, 77, 79] may suggest that this hormone is rapidly catabolized in the fish gonad and may still exist in the fish blood in different forms (glucuronated, sulfonated or reduced) [80–82] and it is not detected by the techniques used for the free steroids. Recent studies suggest that this hormone may play a role in stimulating meiosis or may be released into the water to act as a pheromone [79, 83, 84]. Therefore, in contrast to T and 11-KT, it cannot be considered as a trustworthy indicator of reproductive stage of development in male greater amberjack. Moreover, circulating 17,20β-P levels significantly increased in response to handling stress in the black bream *Acanthopagrus butcheri* [85] and in the greenback flounder *Rhombosolea tapirina* [86] and a positive correlation between cortisol and 17,20β-P was found in sexually mature silver seabream *Pagrus auratus* [87]. The increase of 17,20β-P plasma levels observed in captive-reared greater amberjack with regressed testes in the present study, could then be associated to the handling stress due to sampling operations.

In female greater amberjack in the present study, an increase of T, E_2_ and 17,20β-P from the EARLY to the ADVANCED phase was observed both in wild and captive-reared fish, followed by a significant decrease only of T in the SPAWNING phase, and E2 of captive fish during the SPAWNING phase. A similar pattern was found in another study of wild females sampled in the Mediterranean Sea with the same levels of T and E_2_ or 5-fold lower levels of 17,20β-P [29]. In fact, in the present study T seemed to be elevated only in the ADVANCED phase and dropped at SPAWNING, whereas E_2_ remained high during SPAWNING in wild fish, in agreement with the other study on wild greater amberjack, as high plasma E_2_ levels are essential for the recruitment of new batches of oocytes in fish with asynchronous ovarian development [29]. On the contrary, E_2_ levels dropped during SPAWNING in captive fish in the present study, as ovaries in these females were regressed. As far as 17, 20β-P is concerned, it increased during the ADVANCED phase and remained high during SPAWNING in both wild and captive fish. This hormone is known to be essential for oocyte maturation in different fish species, especially during the later stage of oocyte maturation, which includes germinal vesicle breakdown and yolk globule coalescence [88], a fact that has been confirmed also for greater amberjack and other related species [29, 79, 89]. On the other hand, E_2_ plasma levels of captive-reared greater amberjacks kept in sea pens in Japan were less than 1.2 ng ml^-1^ [90] comparing to 1.95 ng ml^-1^ in this study. However, the small size and the young age (3–4 years) of the sampled Japanese fish should be taken into consideration. The simultaneous elevated T, E_2_ and 17,20β-P plasma levels that were observed during the ADVANCED phase in the present study are typical of an asynchronous spawner such as the greater amberjack [91–93], while the lower sex steroid levels of the captive-reared females are typical of the reproductive dysfunctions observed in captivity [94–96].

Altogether, the comparative analysis of GSI, histological observations and sex steroid plasma levels in the present study indicate a severe adverse effect of confinement in captivity on greater amberjack reproductive axis, with consequent gametogenesis impairment. Differences between wild and captive fish, both histological and in plasma sex steroid levels, were only found in the ADVANCED and the SPAWNING phases and not in the EARLY phase, except for the 17,20β-P in females, stressing the differences in the achievement of maturation between wild and captured fish. More particularly, in captivity the EARLY phase seemed to start correctly, with testes showing all spermatogenetic stages along with luminal spermatozoa and ovaries containing oocytes entering vitellogenesis. In this phase, steroid hormones of captive fish were similar to those of wild individuals. The negative effects of confinement became glaring during the supposed ADVANCED phase, perhaps because the fish sampled at this and the following sampling, had already been manipulated once, during the sampling for the EARLY phase, as they were kept together in the same sea cage. During the ADVANCED phase, when wild greater amberjacks were already in spawning condition, in captive-reared females sex steroid plasma levels showed only a slight increase, insufficient to further support vitellogenesis, leading to an extensive oocyte atresia that prevented any further oocyte development. In captive-reared males, the low steroid levels observed in the EARLY phase decreased further, leading to the precocious cessation of spermatogenic activity.

The reason we believe that this dysfunction might have been caused, at least partly, due to the repeated sampling that the fish underwent in this study, is that in a parallel investigation, another captive-reared stock, maintained in almost identical conditions, reached final stages of gametogenesis and produced fertilized eggs upon stimulation with spawning inducing hormones (C.C.M., unpublished data). The latter stock was manipulated only once in mid-June (between the ADVANCED and SPAWNING samplings) in order to induce spawning, so the husbandry manipulations of the stock used in the present study could have caused/enhanced the reproductive dysfunction. This leads us to suggest that Mediterranean greater amberjack maintained in captivity should not be handled after the onset of gametogenesis, in order to prevent any stress-induced cessation of reproductive development. An easier adaptability of greater amberjack from the eastern Atlantic to captive conditions is reported by other studies [12, 97], who reported natural and hormonally-induced spawning, respectively, of wild caught individuals reared in tanks. Incidentally, the eastern Atlantic and Mediterranean greater amberjack populations are genetically different [98], and it is conceivable that these genetic differences may influence or be responsible for the variable and unpredictable response of greater amberjack from the Mediterranean Sea to confinement in captivity.

Reproductive dysfunctions have been documented in a number of captive-reared fish species, in both females and males. The most common dysfunctions in females are: absence of gonadal development [99]; failure of oocytes to undergo oocyte maturation (also referred to as final oocyte maturation) once vitellogenesis is completed [19, 100, 101]; or absence of spawning [102]. Production of low amount of expressible semen in males [103], as well as reduction of germ cell proliferation and increase of apoptosis [20, 104] has been reported in captive-reared male fish. Atresia of vitellogenic oocyte and failure to undergo oocyte maturation have been attributed to an insufficient pituitary luteinizing hormone (LH) release, and to the consequent steroid withdrawal, occurring in captive conditions [105, 106]. These dysfunctions have been associated to a combination of factors, such as captivity-induced stress, lack of appropriate spawning environment and nutritional deficiencies (an exhaustive review on reproductive dysfunctions, their causes and therapeutic treatments is provided by Zohar and Mylonas [103] and Mylonas et al. [96]).

Considering that liver nutrient mobilization towards the gonads, specifically protein and highly unsaturated fatty acids, plays an important role during gametogenesis, embryo ontogeny and early larval development in marine fish [107], the possibility that essential nutrient deficiency might have played a role in the gametogenesis impairment in the present study has been explored using liver leptin expression and gonad chemical composition as markers. Leptin, the product of the obese gene, is a 16 kDa, 167 amino acid (aa) hormone, consisting of a 21 aa signal peptide and a 146 aa soluble protein [50, 108]. In mammals, leptin is secreted into the bloodstream both as a free protein and as a protein–bound entity, primarily from adipocytes, and acts on the brain to regulate food intake and metabolism [50–55]. In addition to its role in conveying signals of the energy stores to the central nervous system in order to regulate food intake, leptin was also found to interact with the endocrine system to provide critical information about the nutritional status and, therefore, to act as a permissive factor allowing the onset of energy demanding situations such as reproduction [40, 56]. To date, all studies with teleosts have identified the liver as the major site for leptin expression, in contrast to the adipocytes in mammals. In the present study, transcript profiles of liver leptin showed relatively low levels for wild and captive-reared fish during the EARLY and ADVANCED gametogenesis phases, and a dramatic elevation during the SPAWNING phase. Similar patterns, at the protein level, were detected in the freshwater fish burbot *Lota lota* [109], in which the circulating leptin-immunoreactive peptide levels were relatively low prior to and during reproduction, and increased after spawning. It can be hypothesized that the increase in the levels of leptin towards the end of the spawning cycle is a seasonal event helping the fish to recover from the exertion of reproduction, while re-absorbing the gonads and reorganizing the body energy storages. In the present study, no significant differences in leptin expression between wild and captive greater amberjack was found, with the exception of the higher leptin expression levels of captive-reared females in the EARLY period. This is in agreement with minor differences found in gonad proximate composition and seems to indicate that the dietary regime preserved well the energy reserve/nutrient mobilization towards the gonads during the reproductive cycle. However, the testes of captive-reared fish showed different total polar lipid contents, as well as specific lipid classes and fatty acid profiles with respect to wild individuals, clearly reflecting the increased dietary supply of TAG by the commercial diet that contains also vegetables oils as lipid sources. On the contrary, the diet of wild fish is based mainly on fish and cephalopods, which supply both phospholipids and TAG. In particular, testes of captive-reared fish sampled in the EARLY gametogenesis phase displayed a lower PC, PS and, PE, concomitantly to higher TAG, DHA and ARA content compared to the proportions of their wild counterparts. Fish sperm is particularly rich in PE and PS, both of which influence membrane fluidity and male reproductive functions. These molecules contain high levels of di-DHA, molecular species that improves sperm motility and the efficiency of membrane fusion events, such as those taking place between spermatozoa and eggs. Since sperm fatty acid composition depends upon the essential fatty acid content of the broodstock diet [110–112], fertilization success could be affected by lipid profiles. In our study, EPA and LA levels were significantly higher, whereas ARA and DHA were significantly lower in farmed than in wild specimens. The dietary deficiency of ARA and DHA also decreased tissue ARA/EPA and DHA/EPA physiological balances. Similar results related to dietary fatty acid inputs have been reported in several tissues of captive and wild white seabream (*Diplodus sargus*), black seabream (*Spondyliosoma cantharus*), yellowtail (*Seriola lalandi*) and females of greater amberjack at spawning [31, 32, 113–115]. Studies by Asturiano et al. [36] showed that male European sea bass *Dicentrarchus labrax* fed commercial diets enriched in highly unsaturated fatty acids (HUFA) such as DHA and EPA, exhibited more successful reproductive performance in terms of duration of spermiation, total milt production, milt spermatozoa density, and fertilization than fish fed with a non-enriched diet. There is strong evidence that HUFAs, particularly EPA and ARA, via metabolites formed from the cyclooxygenase (COX) and lipoxygenase (LPOX) pathways are involved in steroidogenesis and oocyte maturation in vertebrates [38, 116, 117]. In vitro, ARA stimulates testosterone production in testes and ovaries of several fish species by conversion to prostaglandin [45, 46, 48, 49, 118, 119]. In the present work, captive-reared greater amberjack testes and ovaries contained 40% less ARA than wild fish at EARLY gametogenesis causing strong imbalances of ARA/EPA ratios. Furthermore, expected mobilization of PI as the main source of ARA [107, 120] by the action of phospholipase A2 (PLA2) during steroidogenesis [117] did not occur in the reared fish from early to advanced gametogenesis, presumably correlating with the abnormal trend of steroid levels in captivity. Other phospholipids, including PC, contain high levels of DHA, which is the most relevant essential fatty acid in egg quality [107]. The importance of PC, PE and n-3 HUFA, as well as of DHA/EPA/ARA ratios on gonad development and egg quality has been highlighted by many authors [31, 32, 33–35, 121–124]. In fact, two thirds of the lipid fraction in vitellogenin is made of PC [107] that is also the main phospholipid in mature ovaries and fertilised eggs [125]. Compared to the wild greater amberjack, the total lipids contained in the ovaries of captive-reared fish in the present study also displayed abnormally low ratios of essential fatty acids at the EARLY and ADVANCED phases. According to these results, the formulation of a diet enriched with phospholipids, DHA and particularly with ARA seems to be advisable in order to improve the broodstock general nutritional status.

In conclusion, the occurrence of severe gametogenesis impairment was described in wild caught greater amberjack reared in captivity in the Mediterranean Sea. The observed dysfunctions were possibly related to rearing husbandry (*i*.*e*. multiple handling during the process of gametogenesis), to the lack of natural conditions required for reproductive maturation and/or to nutritional unbalances caused by the artificial diet. An overall improvement of rearing technology, particularly as it relates to husbandry operations (*e*.*g*. fish handling and transferring) together with a better formulation of dietary ingredients, are required to overcome the observed dysfunctions and lay the basis for a sustainable aquaculture of this species.

## Supporting Information

S1 Dataset Biometric data, sex steroids, leptin and gonad biochemical composition of wild and captive-reared greater amberjack. (XLSX)

S1 Fig Partial cDNA and deduced amino acid sequences of greater amberjack leptin. The putative alpha helix domains are specified.
(TIF)

S1 Table Proximate (a) and main fatty acids composition (b) of greater amberjack broodstock diet. (DOC)

S2 Table Similarity of the predicted *Seriola dumerili* ORF to leptin amino acid sequences derived from various Perciformes. (DOCX)
