# Supplementary figures and images for: Comparative Study of Reproductive Development in Wild and Captive-Reared Greater Amberjack Seriola dumerili (Risso, 1810)
Source: PLoS One. 2017 Jan 5;12(1):e0169645. doi: 10.1371/journal.pone.0169645 (PMC5215828; doi:10.1371/journal.pone.0169645)

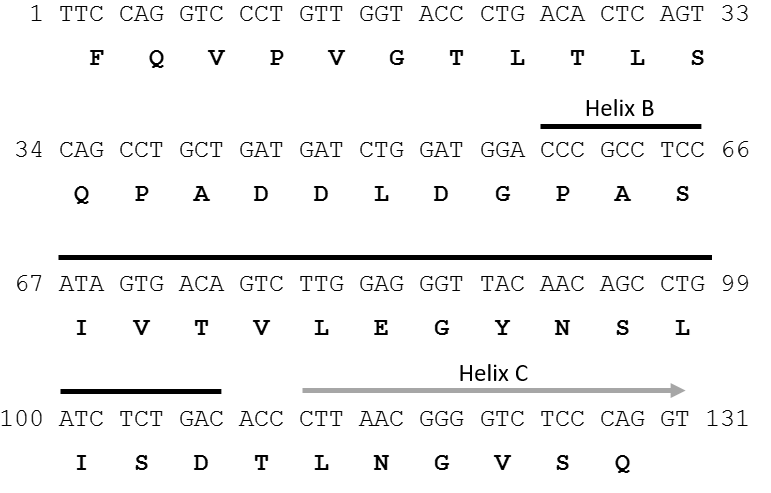

Supplement: S1 Fig — The putative alpha helix domains are specified. (TIF) [file pone.0169645.s002.tif]
