# Supplementary material for: Comparative Study of Reproductive Development in Wild and Captive-Reared Greater Amberjack Seriola dumerili (Risso, 1810)
Source: PLoS One. 2017 Jan 5;12(1):e0169645. doi: 10.1371/journal.pone.0169645 (PMC5215828; doi:10.1371/journal.pone.0169645)
Supplement: S1 Table — (DOC) [file pone.0169645.s003.doc]

**(a) Dietary proximate composition.**

| Moisture (%) | 10.4 ± 0.0 |
| --- | --- |
| TL (%DM) | 18.0 ± 0.4 |
| Prot (%DM) | 55.9 ± 1.4 |
|  |  |

Values expressed as mean ± SE (n=2). TL, total lipids; Prot, proteins.

**(b) Main fatty acid composition of diet (% of total fatty acids).**

| 16:0 |  | 16.9 ± 0.0 |
| --- | --- | --- |
| 18:1^1^ |  | 21.9 ± 0.1 |
| 18:2n-6 |  | 7.7 ± 0.0 |
| 20:4n-6 |  | 1. 0.0 |
| 20:5n-3 |  | 10.2 ± 0.1 |
| 22:6n-3 |  | 11.0 ± 0.3 |
|  |  |  |
| DHA/EPA |  | 1.1 ± 0.0 |
| ARA/EPA |  | 0.1 ± 0.0 |
|  |  |  |

Values expressed as mean ± SE (n=2).

^1^, mainly n-9 isomer. DHA, docosahexaenoic acid, 22:6n-3; EPA, eicosapentaenoic acid, 20:5n-3; ARA, arachidonic acid, 20:4n-6.
