# Supplementary material for: Comparative Study of Reproductive Development in Wild and Captive-Reared Greater Amberjack Seriola dumerili (Risso, 1810)
Source: PLoS One. 2017 Jan 5;12(1):e0169645. doi: 10.1371/journal.pone.0169645 (PMC5215828; doi:10.1371/journal.pone.0169645)
Supplement: S2 Table — (DOCX) [file pone.0169645.s004.docx]

| **Species** | **Amino acid sequence Identity (%)** | | | **NCBI accession number** |
| --- | --- | --- | --- | --- |
| ***Siniperca chuatsi*** | | 90% | ACT98260.1 | |
| ***Lateolabrax japonicus*** | | 88% | AHI85768.1 | |
| ***Epinephelus coioides*** | | 86% | BAI66433.1 | |
| ***Dicentrarchus labrax*** | | 81% | AIY99976.1 | |
| ***Thunnus thynnus*** | | 69% | ADT91717.1 | |
| ***Oreochromis niloticus*** | | 67% | NP 001287979.1 | |
